# Communicating increased driving risk: A study of mature driver expectations

**DOI:** 10.1002/alz70858_106169

**Published:** 2025-12-24

**Authors:** Lindsay McCauley, Kathleen Van Benthem, Chris Herdman, Bruce Wallace, Jocelyn Keillor, Rafik Goubran, Frank Knoefel, Shawn Marshall

**Affiliations:** ^1^ Carleton University, Ottawa, ON, Canada; ^2^ Bruyere Health Research Institute, Ottawa, ON, Canada; ^3^ AGE‐WELL NIH SAM3, Ottawa, ON, Canada; ^4^ National Research Council of Canada, Ottawa, ON, Canada; ^5^ Bruyere Continuing Care, Ottawa, ON, Canada; ^6^ University of Ottawa, Ottawa, ON, Canada

## Abstract

**Background:**

Driving is essential for maintaining mobility, social participation, and independence, yet it relies on cognitive and physical abilities that may decline with age, increasing risks for older adults. Drivers aged 65 and older experience disproportionately higher rates of collisions and fatalities compared to middle‐aged drivers. Many struggle with decisions about adjusting their driving habits or retiring but rarely use existing guidelines. Additionally, few older drivers assess their abilities before mandatory licensing tests, leaving them unaware of potential risks.

**Method:**

This study explores the needs of older drivers to help them understand their collision risks and plan for driving retirement. A nationwide survey of Canadian drivers aged 55+ was conducted with ethics approval from Carleton University and the Bruyère Health Research Institute. The survey received 386 responses (average age: 75) and examined driving habits, perceptions of vehicle data collection, and preferences for receiving feedback on driving safety.

**Result:**

Three main themes emerged from the data: empowerment, privacy, and personalization. Results showed that 65% of drivers already use in‐vehicle safety technologies, but half required more information before adopting new tools. Participants expressed interest in features like driving behaviour reports, personalized safety tips, and historical driving trends. Privacy and security concerns (68%) and technology accuracy (52.4%) were barriers to adoption, while ease of use (65%), strong privacy protections (56.4%), and proven reliability (55.9%) were key motivators. Respondents preferred to receive feedback on demand (50.4%) or monthly (32.2%) via email (53%) or app‐based platforms (45.2%). Most participants preferred sharing feedback with spouses (51.6%), while fewer were comfortable sharing it with children (35%) or healthcare providers (16.2%).

**Conclusion:**

These findings suggest that driving feedback technologies could help older drivers monitor their abilities and engage in evidence‐based discussions with healthcare providers. Objective data enables healthcare providers to address sensitive topics, like declining driving ability, with actionable insights, easing emotional challenges. Prioritizing empowerment, privacy, and personalization ensures trusted, user‐focused tools that support self‐monitoring, informed decisions, and collaboration, promoting safer driving and sustained independence.

